# Acceleration of Molecular Simulations by Parametric Time-Lagged tSNE Metadynamics

**DOI:** 10.1021/acs.jpcb.3c05669

**Published:** 2024-01-18

**Authors:** Helena Hradiská, Martin Kurečka, Jan Beránek, Guglielmo Tedeschi, Vladimír Višňovský, Aleš Křenek, Vojtěch Spiwok

**Affiliations:** † Department of Biochemistry and Microbiology, University of Chemistry and Technology Prague, Technická 3, Prague 6 166 28, Czech Republic; ‡ Institute of Computer Science, Masaryk Univerzity, Šumavská 416/15, Brno 602 00, Czech Republic

## Abstract

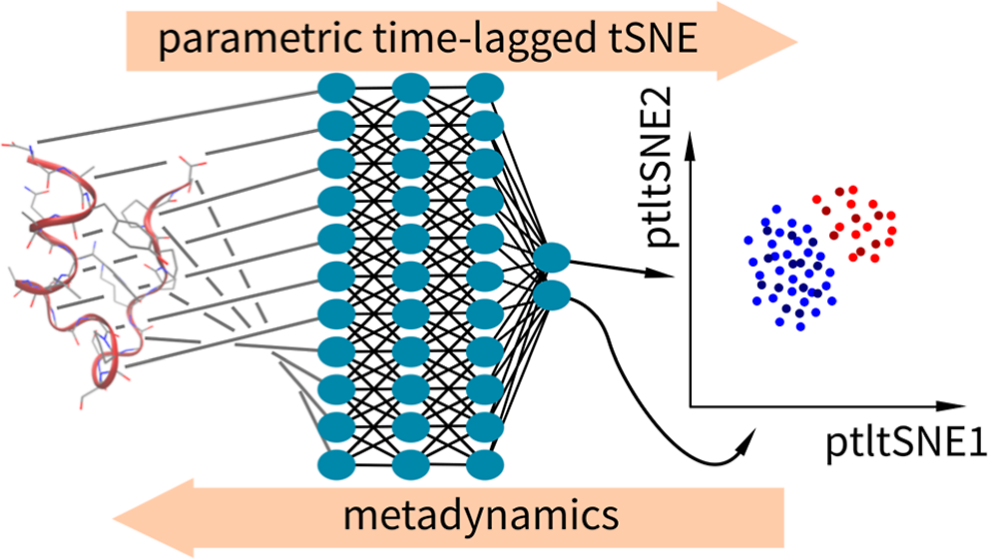

The potential of molecular simulations is limited by their computational costs. There is often a need to accelerate simulations using some of the enhanced sampling methods. Metadynamics applies a history-dependent bias potential that disfavors previously visited states. To apply metadynamics, it is necessary to select a few properties of the system—collective variables (CVs) that can be used to define the bias potential. Over the past few years, there have been emerging opportunities for machine learning and, in particular, artificial neural networks within this domain. In this broad context, a specific unsupervised machine learning method was utilized, namely, parametric time-lagged t-distributed stochastic neighbor embedding (ptltSNE) to design CVs. The approach was tested on a Trp-cage trajectory (tryptophan cage) from the literature. The trajectory was used to generate a map of conformations, distinguish fast conformational changes from slow ones, and design CVs. Then, metadynamic simulations were performed. To accelerate the formation of the α-helix, we added the α-RMSD collective variable. This simulation led to one folding event in a 350 ns metadynamics simulation. To accelerate degrees of freedom not addressed by CVs, we performed parallel tempering metadynamics. This simulation led to 10 folding events in a 200 ns simulation with 32 replicas.

## Introduction

In the past decades, numerous studies have demonstrated that the conformational dynamics of biomolecules is equally important as their 3D structures. Computational modeling of biomolecular dynamics, in particular the method of molecular dynamics (MD) simulation, has become an important alternative and complement to experimental methods.^1,2^

The impact of MD simulations has always been limited by their high computational costs caused by a small step and a huge number of interatomic potentials evaluated in every step. For this reason, it is possible to simulate nanosecond to microsecond time scales, but longer time scales are still not routine as they require either high computational power in terms of numbers of CPUs and GPUs, or special purpose hardware.

Nanosecond to microsecond timescales often do not allow for sampling of all important states of the studied system. For this reason, numerous extensions of MD have been developed to enhance sampling so that important states can be sampled and their densities can be predicted by short simulations. In this work, we use metadynamics.^3^ This method enhances sampling by “flooding” local energy minima with an artificial history-dependent bias potential.

The bias potential in metadynamics and many other enhanced sampling methods is a function of a few predefined descriptors of the state of the system known as collective variables (CVs). Choice of CVs is critical to the efficiency of sampling enhancement, especially in complex biomolecular systems.

Any set of CVs reduces the dimensionality of the studied system because it describes a system with high-dimensional Cartesian coordinates **x** using low-dimensional CVs **s**. Therefore, general dimensionality reduction techniques (**x** → **s**) are predisposed to work as generally applicable methods to design CVs. Indeed, numerous linear and nonlinear dimensionality reduction techniques have been tested as CVs to map high-dimensional conformations into low-dimensional maps and to drive sampling of this map by biased simulations.^4−11^

The disadvantage of linear dimensionality reduction methods is that the motions of atoms in molecules are nonlinear. Therefore, nonlinear dimensionality reduction methods are likely to provide a better description of the system compared to linear methods with the same number of CVs. tSNE is a successful nonlinear dimensionality reduction method popular in bioinformatics, image analysis, and other fields.^12,13^ It has also been applied successfully to analyze molecular simulation trajectories.^14−16^

One of the reasons behind the success of tSNE in different fields of science is its focus on proximity (distance transformed by the Gaussian function) rather than distance of high-dimensional points. Many linear and nonlinear dimensionality reduction methods have been designed so that they accurately reproduce the distances between high-dimensional points **x** in a low-dimensional space. However, the development of dimensionality reduction methods has shown that the proximity of the points is often more important than their distance. It is more important to cluster together similar points (e.g., gene expression profiles of patients with the same diagnosis), instead of reproducing distances of distant points (gene expression profiles of patients with completely different diagnoses). For this reason, tSNE works with proximities of points in a low-dimensional space rather than distances.

tSNE starts with a set of points **x** in a high-dimensional space. The number of points is *N* and their dimension is *M*. In the molecular world, this could be a trajectory with *N* time frames and *M* Cartesian coordinates (*M* = number of atoms × 3). The method first computes the proximities *p*_*ij*_
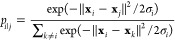
1
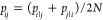
2

The variable σ_*i*_ is the bandwidth of the Gaussian kernels. It is controlled by the perplexity parameter (see ref (13) for details). Next, points **s** in the low-dimensional space are initially estimated by linear dimensionality reduction of **x**. The proximity in the low-dimensional space *q*_*ij*_ is calculated as
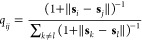
3

Finally, the values of **s** are optimized to reach the best agreement of *p*_*ij*_ and *q*_*ij*_, expressed as the Kullback–Leibler divergence
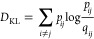
4

Unfortunately, the main disadvantage of most nonlinear dimensionality reduction methods, including tSNE, is the fact that it is possible to analyze *N* high-dimensional structures and obtain their low-dimensional embeddings, but it is not possible to calculate low-dimensional embeddings for a new out-of-sample (*N* + 1-th) structure. Furthermore, the application of bias forces in the direction of a CV requires the calculation of the first derivative of the CV with respect to the Cartesian coordinates of the atoms. This is also not possible for most nonlinear dimensionality reduction methods including tSNE.

This problem can be solved by an application of a variant of tSNE called parametric tSNE.^17^ The original tSNE finds low-dimensional embeddings **s** of data **x** to get the best agreement (Kullback–Leibler divergence) between similarities of **x** (defined as *p*_*ij*_) and similarities of **s** (defined as *q*_*ij*_). Instead of direct optimization of the values of **s**, parametric tSNE uses a neural network to calculate **s** from **x** and optimizes the parameters (weights and biases) of this neural network. The trained neural network can be used to calculate a low-dimensional embedding for any out-of-sample structure. The analytical derivatives of **s** with respect to **x** can also be easily calculated because neural networks are designed to be trained using the backpropagation algorithm, which requires automatic differentiation.

An important limitation of general dimensionality reduction methods is the fact that they highlight the most intensive motions in the system (modes with the highest variance) rather than the slowest ones. However, the best performance of the enhanced sampling methods is achieved when slow motions are accelerated. Conventional methods of dimensionality reduction may lead to acceleration of intensive but fast motions such as motions of protein loops or flexible N-terminal or C-terminal tails. It is possible to emphasize slow motions and weaken intensive but fast motions using a time lag. This is applied in time-lagged independent component analysis (TICA)^18,19^ or time-lagged tSNE.^16^

In this work, we combined the advantages of several approaches presented above, namely, metadynamics, tSNE, neural network, and time lag, to calculate the low-dimensional embedding **s** from coordinates **x**. We applied a parametric time-lagged tSNE to calculate CVs and to accelerate the folding and unfolding of the tryptophan cage (Trp-cage) miniprotein.

## Methods

The code for the parametric time-lagged tSNE is available online at https://github.com/spiwokv/ptltsne, based on Keras,^20^ TensorFlow,^21^ and PyTorch.^22^ Lag time is introduced as described in the article introducing a time-lagged tSNE.^16^ Briefly, it uses the algorithm inspired by AMUSE^23^ to obtain a time-lagged high-dimensional representation, followed by a dimensionality reduction by tSNE. In detail, the *M* × *M* covariance matrix is calculated from centered coordinates fitted to a reference structure. The coordinates are transformed onto eigenvectors of the matrix normalized by the roots of its eigenvalues. The resulting flattened normalized projections are used to calculate the time-lagged covariance matrix. This matrix is symmetrized by calculating means of above- and below-diagonal terms (**C**_sym_^**Y**^ = 1/2(**C**^**Y**^+(**C**^**Y**^)^*T*^)). Eigenvectors of this matrix are expanded by the roots of its eigenvalues. Finally, the trajectory is projected onto these eigenvectors. The resulting projections are analyzed by tSNE.

The 208 μs trajectory of the folding and unfolding of the Trp-cage, used as training data, was provided kindly by D.E. Shaw Research.^24^

Metadynamics simulations were performed in Gromacs-2021.4^25^ patched with Plumed-2.8.0.^26,27^ Parallel tempering metadynamics was performed in Gromacs-2021 patched by Plumed-2.9.0 with the PyTorch module (https://github.com/kurecka/plumed2/tree/uvt_extensions, different implementation than in ref (28)). This code makes it possible to use an artificial neural network in PyTorch^22^ in Plumed (see Supporting Information for details).

The structure of the Trp-cage (sequence DAYAQWLKDGGPSSGRPPPS)^29^ was obtained from the Protein Databank (PDB-ID 2JOF, the first model).^30^ It was modeled using the Amber99SB-ILDN force field^31^ for the protein and SPC-E model for water^32^ (2540 water molecules in metadynamics and 1616 for parallel tempering metadynamics). A smaller box size was used for parallel tempering to increase the relative fluctuation of potential energy, thus increasing replica exchange probability. One chloride was added as a counterion to each system.

The energy of the systems was minimized by the steepest-descent method. Next, they were equilibrated by 100 ps simulation in a NVT (constant number of particles, volume, and temperature) and 100 ps simulation in a NPT (constant number of particles, pressure, and temperature) ensemble with harmonic restraints applied on non-hydrogen atoms of the protein. The simulation step was set to 2 fs. Bonds involving hydrogen atoms were constrained by the LINCS algorithm.^33^ Temperature and pressure were controlled by the Parrinello-Bussi^34^ and Parrinello–Rahman^35^ algorithms, respectively. Temperature was set to 300 K in metadynamics without parallel tempering. The pressure was set to 1 bar.

Well-tempered^36^ 1.5 μs metadynamics was performed with two ptltSNE CVs or two ptltSNE CVs with α-RMSD CV. The bias potential was formed by the sum of Gaussian hills added every 1 ps. The width of a hill was 1 in the direction of both ptltSNE CVs and 0.2 in the direction of α-RMSD. The height was set at 0.5 kJ/mol. The bias factor was set to 8. These settings (widths relative to CV ranges) follow recommendations for metadynamics^37^ and well-tempered metadynamics.^36^

Parallel tempering metadynamics^38^ was performed with the ptltSNE CVs (without α-RMSD) in the NVT ensemble (200 ns per replica). We selected 32 temperatures of replicas, namely 278, 285, 292, 299, 307, 314, 322, 330, 338, 347, 355, 364, 373, 382, 392, 402, 412, 422, 432, 443, 454, 465, 477, 489, 501, 514, 526, 539, 553, 566, 581, and 595 K. These values were selected to cover biological as well as elevated temperatures. They are exponentially distanced, which usually provides efficient replica exchange at all temperatures. Replica exchange attempts were made every 500 steps (1 ps).

The visualization of metadynamics results was performed using metadynminer and metadynminer3d packages^39^ or ad hoc scripts.

Raw data (input files and scripts, trajectories without water, and other files) are available online at Zenodo^40^ (DOI: 10.5281/zenodo.8246334, DOI: 10.5281/zenodo.8246298), GitHub (https://github.com/spiwokv/ptltsne-visualizations) and Plumed Nest^27^ (https://www.plumed-nest.org/eggs/23/032/).

## Results

### Parametric Time-Lagged tSNE

The trajectory of Trp-cage folding and unfolding was analyzed using ptltSNE. Analysis was performed on non-hydrogen atoms on 10,439 samples of the 208 μs trajectory. There are multiple hyperparameters for ptltSNE. It is possible to set the lag time, number, and size of layers of the artificial neural network, the activation functions, the batch size, the shuffle time, and the number of training epochs. We tested different combinations of hyperparameters using a trial and error approach, and we selected the settings described below. It is possible to choose hyperparameters with some modern hyperparameter tuning tools; however, the trial and error approach gave us the opportunity to test the functionality of the software for a wide range of hyperparameters. Nevertheless, the experimental application of a tuning tool confirms the trial and error selection to be correct (data not shown).

The Cartesian coordinates were centered on the reference structure and scaled to be in the range of 0–1. Next, they were transformed to introduce the lag time, as defined above. A lag time of 2 (in the number of frames, i.e., 40 ns) was used. The transformed coordinates were used as input. These signals were processed by a feedforward neural network with three hidden layers, each with 256 neurons with the tanh activation function, without biases. In the end, there was a two-dimensional linear output with biases. Perplexity was set to 30.

The training was carried out by 10,000 backpropagation epochs using the ADAM algorithm with batches of size 1024 (shuffle period 10).

**Figure 1:**
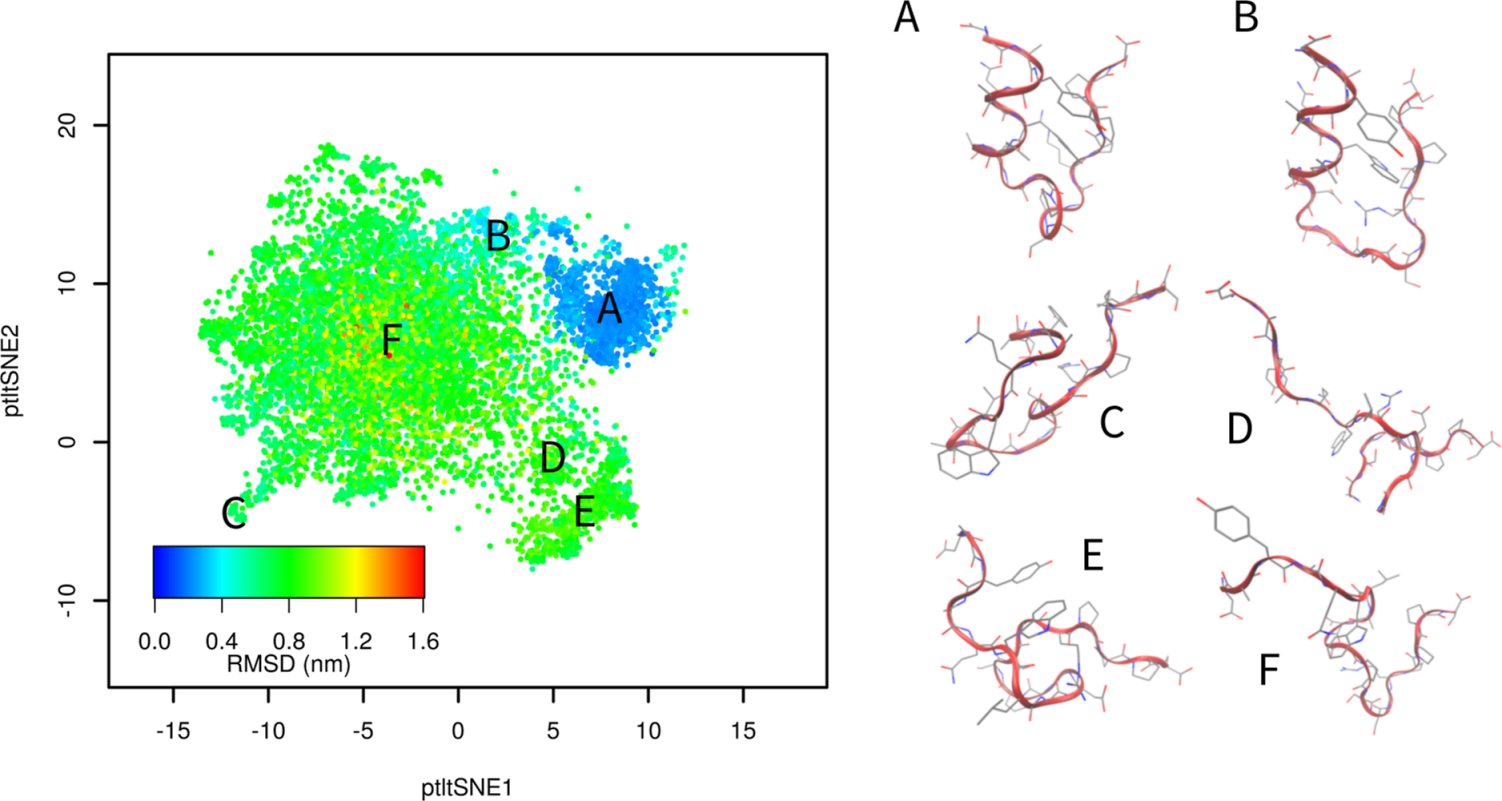
Dimensionality reduction of the Trp-cage trajectory by ptltSNE. Each point represents one snapshot of the training trajectory colored by RMSD. The simulation started from the folded state. The parameters were the following: 10,000 epochs, lag time 2, 3 hidden layers, each with 256 neurons, tanh activation function, batch size 1024, and shuffle interval 10. The plot shows a low-dimensional representation of the data from the Trp-cage trajectory, and the axes are the two CVs obtained by ptltSNE. The data points are colored based on calculated RMSD from the native structure. Representative structures of the Trp-cage for several parts of the plot are also presented (A–F).

The results of ptltSNE are shown in Figure 1. Analogous figures colored by time are available in the Supporting Information (Figures S1–S11). Each structure sampled in the 208 μs trajectory is represented as *a* point. Their distribution shows some characteristics of tSNE, time-lagged tSNE, and parametric tSNE. That is, points are clustered into multiple clusters, and within each cluster, they are evenly distributed. This is typical for tSNE, where an even distribution of points is ensured by the perplexity. The central cluster (F) represents unfolded structures. These structures are highly variable (i.e., different in RMSD values). This is the result of time lag, which causes short-lived structures to cluster together. A similar pattern was observed for time-lagged tSNE.^16^ Finally, the separation of clusters in ptltSNE is not as distinct as that in tSNE or time-lagged tSNE. This can be explained by the fact that a relatively small feedforward neural network was used to transform high-dimensional to low-dimensional data. It was necessary to make a trade-off between the separation of clusters and the performance of the neural network. Transformations of forces from CV space to the space of Cartesian coordinates must happen in every step of metadynamics, so a large neural network would decrease the simulation performance, even with an efficient neural network implementation.

Figure 1 shows the structural representation of the selected clusters. The cluster (A) corresponds to the folded structure. The cluster (B) corresponds to a partially folded structure. The figure follows the pattern observed in time-lagged tSNE analysis^16^ with the unfolded structure (F) in the center and the folded (A) and other long-living structures (C–E) located around the central cluster. These long-lived structures (“kinetic traps”) are stabilized by hydrogen bonds. The unfolded structure (F) does not show hydrogen bonds.

### Metadynamics

The set of ptltSNE coordinates was applied as CVs in metadynamics. Well-tempered^36^ 1.5 μs metadynamics was performed with two ptltSNE CVs.

**Figure 2:**
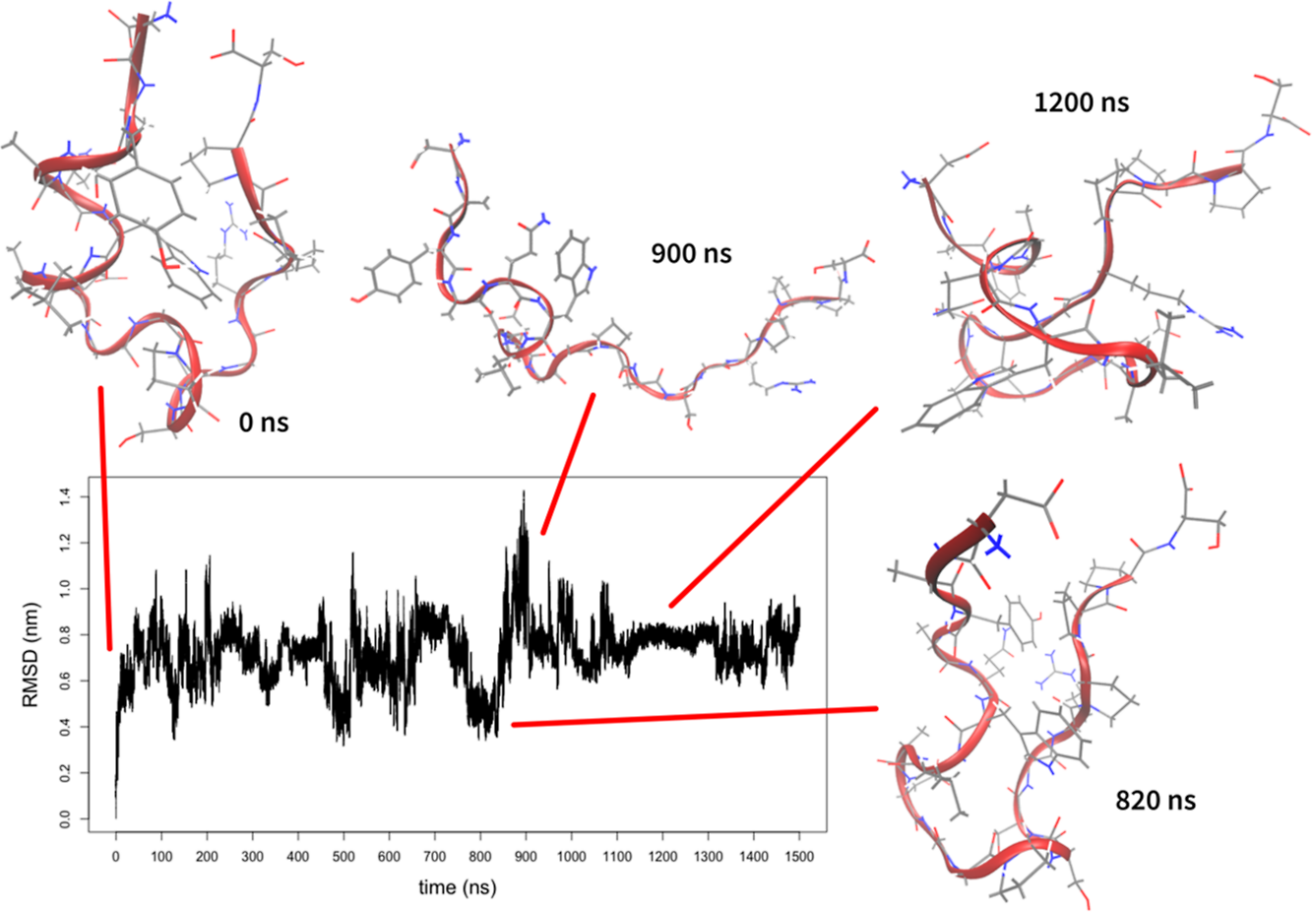
Progress of metadynamics simulation of the Trp-cage with CVs obtained by ptltSNE. The plot shows changes of RMSD from the native structure over time. Several representative structures are shown.

**Figure 3:**
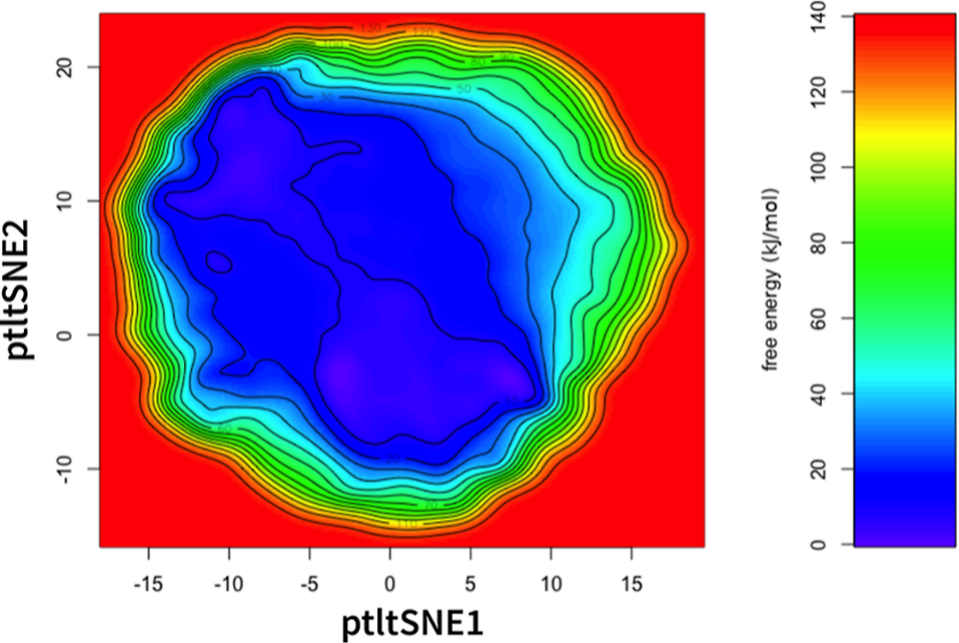
Free energy surface from metadynamics simulation of Trp-cage with CVs obtained by ptltSNE. Due to the absence of the folding event, this free energy surface does not reflect folding equilibrium.

The results are depicted in Figure 2. The RMSD profile shows that after unfolding, the system did not fold back to the native structure. There were multiple visits of structures close to the native state with RMSD from the native structure of approximately 0.4 nm but missing the key helix. This was reflected in the absence of the minima at the point corresponding to the folded state on the free energy surface (Figure 3).

The possible explanation for the absence of folding in the metadynamics simulation could be the fact that the CVs tested cannot efficiently accelerate the formation of α helices. To address this issue, we performed a 1.3 μs metadynamics simulation with three CVs, namely with two ptltSNE CVs (the same as in the previous simulation) and α-RMSD.^41^ This CV has been developed to accelerate the formation of α helices.

**Figure 4:**
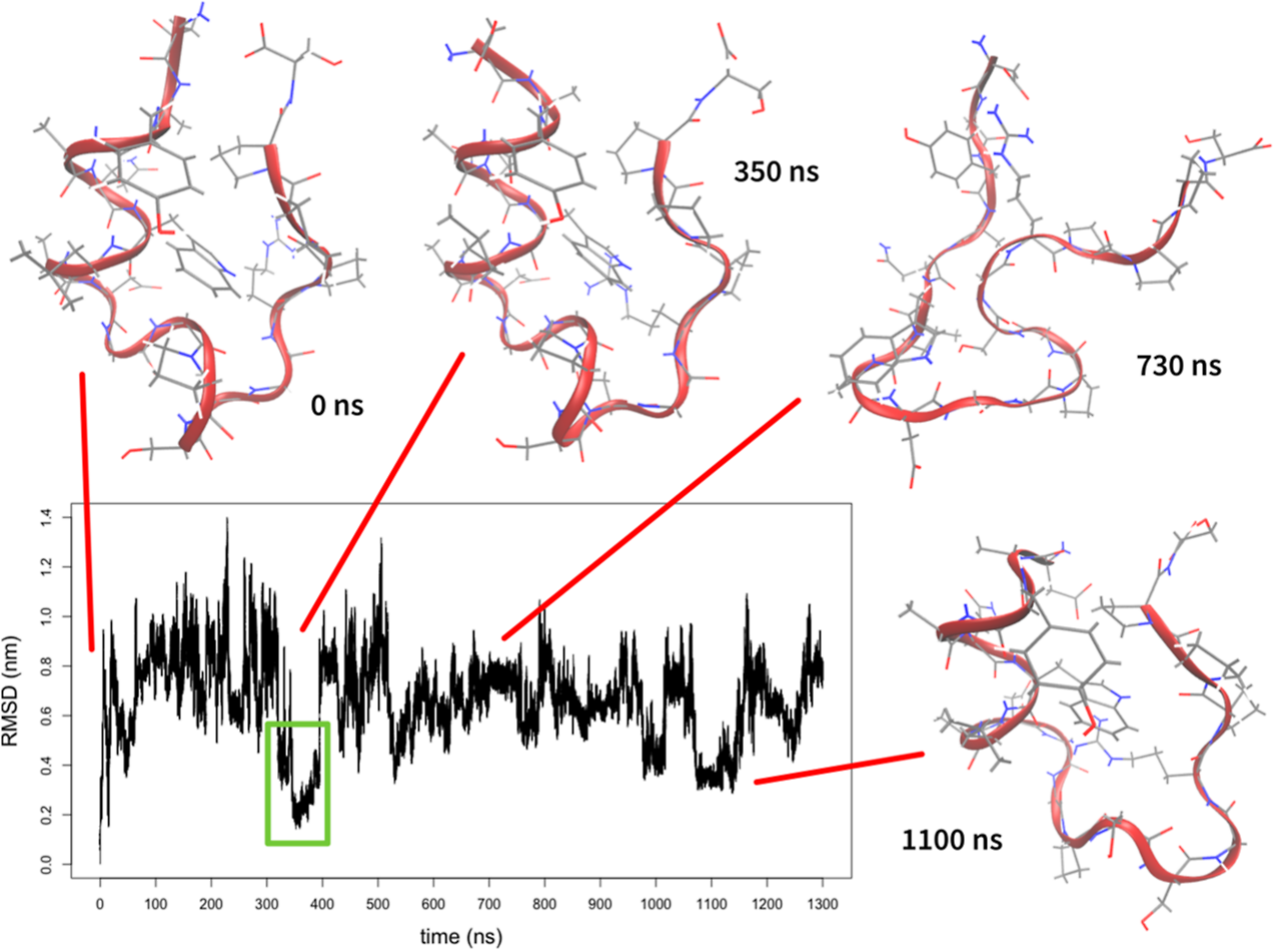
Progress of metadynamics simulation of Trp-cage with CVs obtained by ptltSNE and α-RMSD. The plot shows changes in RMSD from the native structure over time (the folding event is highlighted by the green frame). Several representative structures are shown.

**Figure 5:**
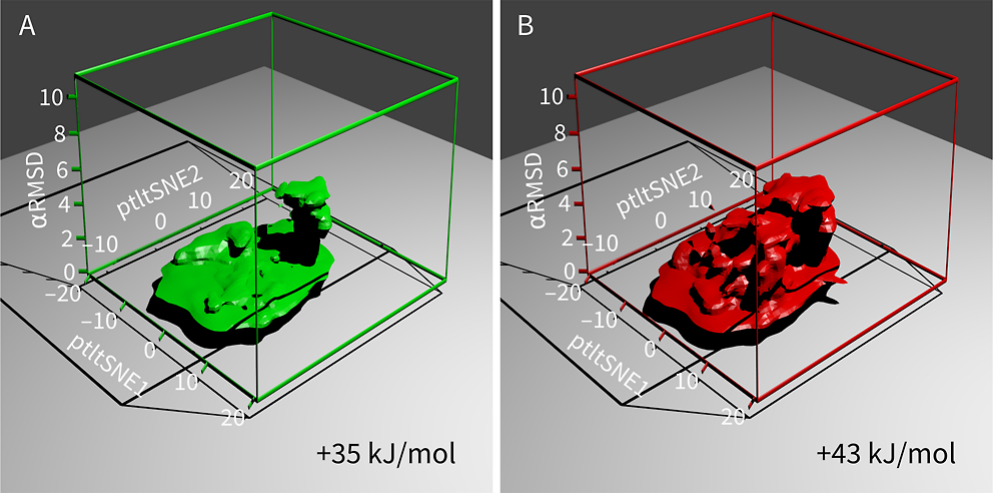
Free energy surfaces calculated by metadynamics simulation of Trp-cage with CVs obtained by ptltSNE and α-RMSD. Isosurfaces at +35 (A) and +43 (B) kJ/mol (relative to the global minimum) are presented. A lobe with high values of α-RMSD, corresponding to the folded state, is visible in panel (A).

In the metadynamic simulation with the ptltSNE and α-RMSD CVs, we observed folding after approximately 350 ns (Figure 4). The free energy surface is shown in Figure 5. Because we used three CVs, the free energy surface is three-dimensional. The isosurface shows that there are many nonhelical states of the protein at the bottom of the 3D plot (low α-RMSD) and a single native minimum with helix (high α-RMSD). The native structure is visible as a vertical lobe in Figure 5A. Figure 5B shows that there are also multiple high-energy states containing α helices. Isosurfaces at other levels are provided as interactive visualizations (https://github.com/spiwokv/ptltsne-visualizations).

It is clear that a free energy surface calculated from a simulation with a single folding event cannot be accurate because of the lack of data. For this purpose, we tested the ptltSNE CVs (without α-RMSD) in 200 ns parallel tempering metadynamics.^38^ This method combines parallel tempering^42^ with metadynamics to accelerate slow motions that are not controlled by CVs. We selected 32 temperatures for replicas from 278 to 595 K.

**Figure 6:**
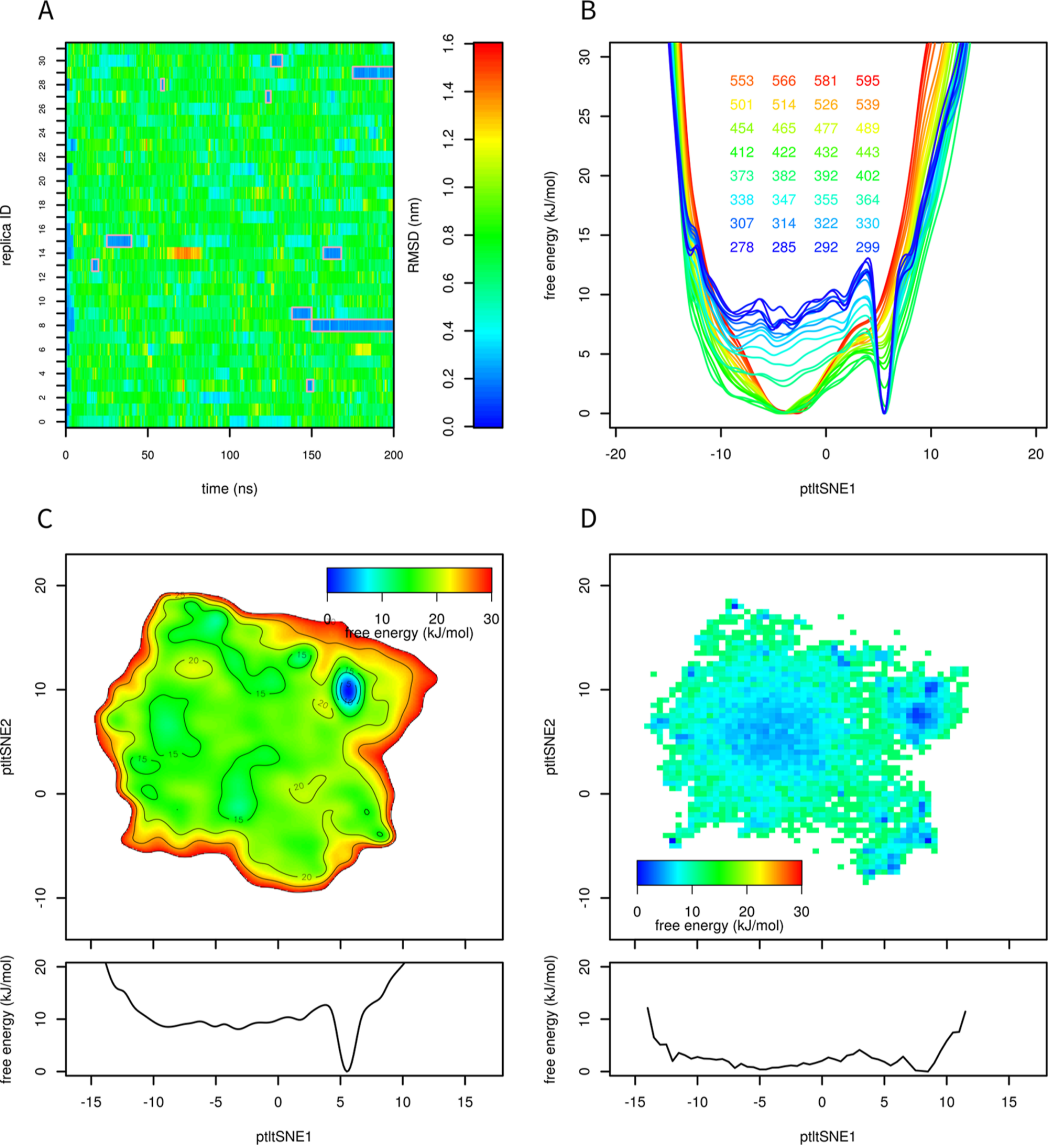
Parallel tempering metadynamics. Root-mean-square deviation from the native structure in demultiplexed trajectories is depicted by the color (A). Ten folding events can be seen in the figure (folded states are highlighted by magenta frames). One-dimensional free energy surfaces at different temperatures are depicted by different colors (B). Free energy surface at 292 K was calculated from the bias potential (C). Free energy surface at 290 K calculated from the training 208 μs trajectory^24^ (D).

We observed 10 folding events (Figure 6A) in demultiplexed (demuxed) trajectories of parallel tempering metadynamics (200 ns for each replica). In comparison, there were two folding events in a standard parallel tempering simulation of the same length (results not shown; see the Supporting Information sets).

For a better comparison of free energy surfaces at different temperatures, we converted 2D surfaces to 1D. This was done by conversion of the 2D free energy surface to the 2D probability profile, followed by integration with respect to one CV and conversion back to free energy. These 1D free energy profiles are shown for all temperatures in Figure 6B. Clearly, free energy surfaces at low temperatures show a deep minimum corresponding to the folded state (ptltSNE1 at 6–7), whereas free energy surfaces at high temperatures are broad with a single minimum at the center.

The free energy surface at 292 K (Figure 6C) was compared with the free energy surface obtained from the training (Figure 6D, 290 K). These free energy surfaces were obtained by different methods (scaled negative image of the metadynamics bias potential vs conversion of a 2D histogram to the free energy surface); therefore, they greatly differ in smoothness. However, both free energy surfaces are very flat (except for the peak in the folded state in Figure 6C). Therefore, despite different methods, force fields, lengths of simulations, and slightly different temperatures (292 vs 290 K), they are in very good agreement.

A common misconception in molecular modeling is the estimation of relative populations of states solely by comparing the depths of the free energy minima. This approach neglects the effect of the widths of the free energy minima. To avoid this, it is necessary to account for different widths of the free energy basins.^43^ This is especially important for tSNE (as well as the parametric and time-lagged variants) because of its tendency to uniformly distribute data points in low-dimensional space. The uniform distribution of points in the low-dimensional space makes the resulting free energy surface flat. This makes the effect of the widths of the free energy minima important.

**Figure 7:**
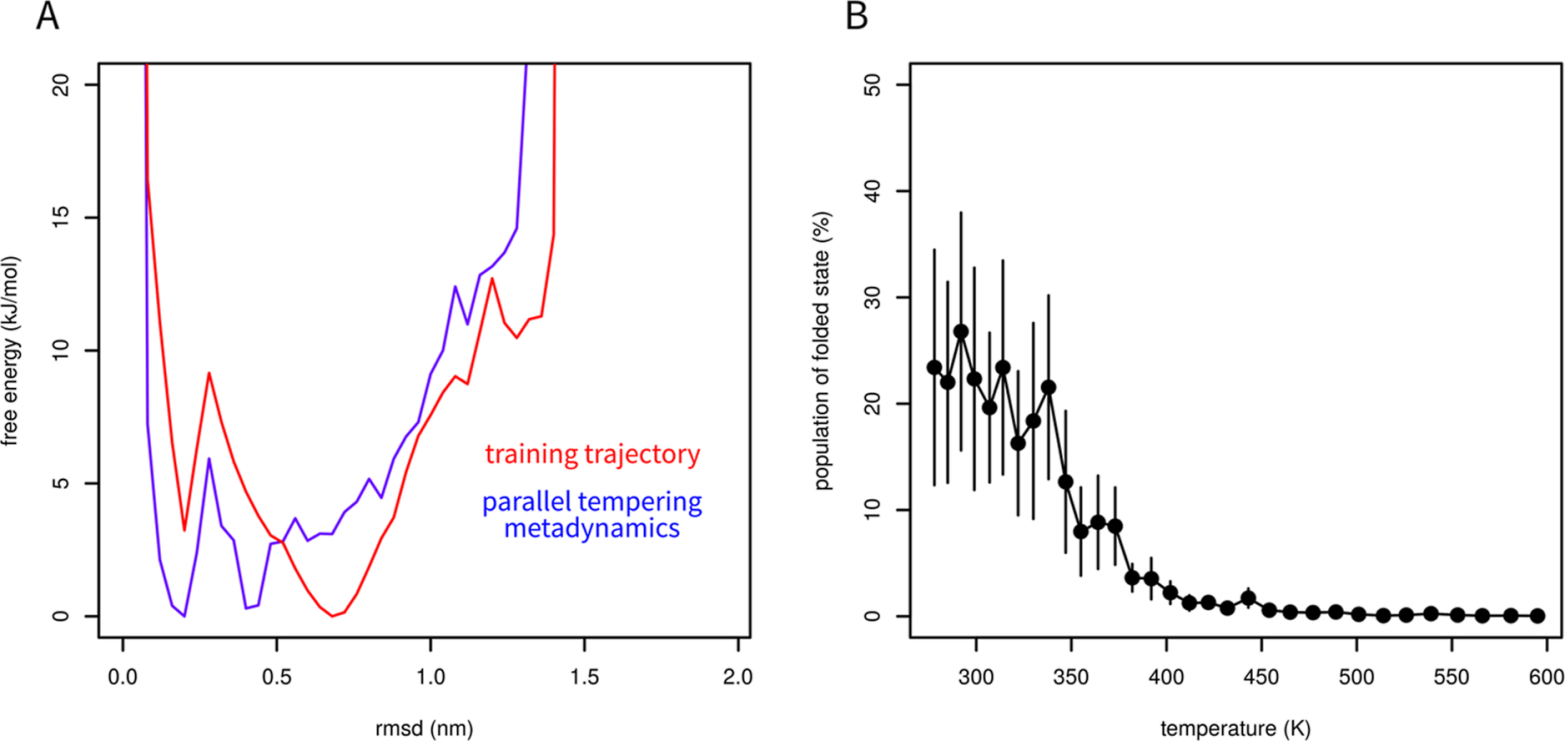
Reweighting of parallel tempering simulation. The free energy surface at 292 K (blue) is compared to the free energy calculated from the reference 208 μs trajectory (red, 290 K). Both free energy surfaces were calculated with RMSD from the native structure as the collective variable (A). Fraction of folded state as a function of temperature (mean ± SEM) predicted by reweighting the results of parallel tempering metadynamics (B).

Structural interpretation of ptltSNE CVs is not intuitive; therefore, we recalculated the free energy surface with a new CV, namely, RMSD from the native structure. The new free energy profile can be obtained by a reweighting procedure, i.e., by estimating unbiased probabilities from biased simulations. The results of parallel tempering metadynamics were reweighted by umbrella sampling reweighting^44^ with a Tiwary–Parrinello correction for the time dependence of the metadynamic bias potential.^45^ RMSD less than 0.35 nm was considered as the folded state. The first 1 ns was discarded as an equilibration. The results expressed as the free energy surface in the space of RMSD and the fraction of the folded state are shown in Figure 7. The free energy surface calculated from the training trajectory is provided in Figure 7A (note that these simulations differ in force field and slightly in temperature). The error bars in Figure 7B represent the standard error of the mean calculated in ten equally sized time windows.

The folded state accounts for tens of percent at low temperatures. The fraction of the folded state decreases with the temperature, as expected, except for noise. Its population is negligible at temperatures higher than 400 K.

**Figure 8:**
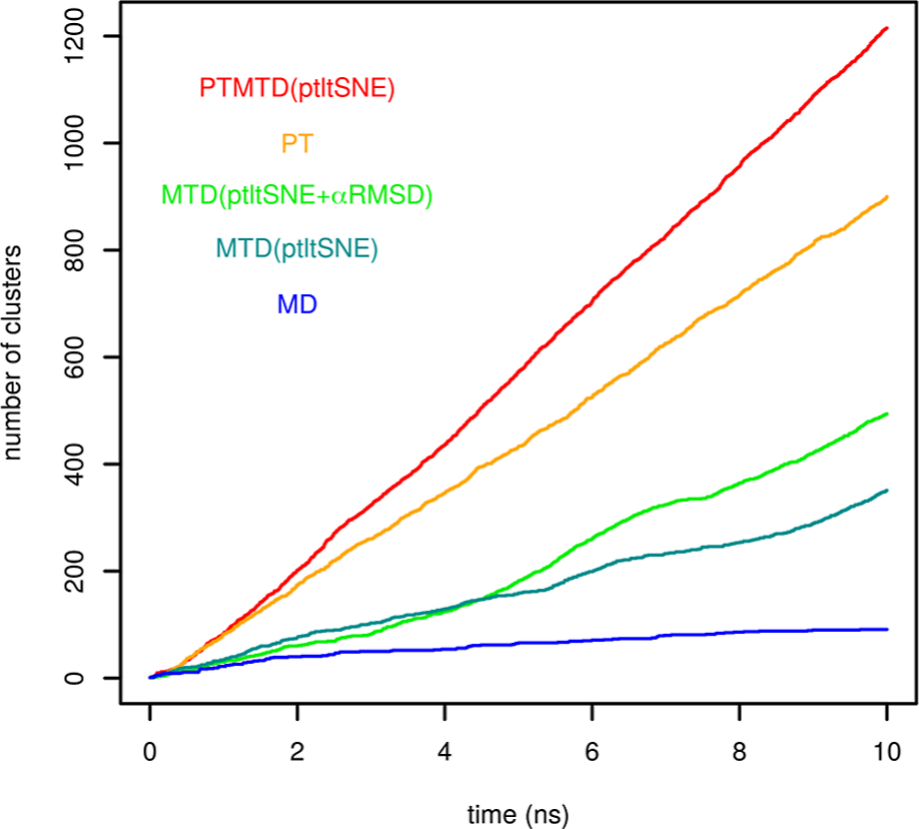
Evolution of the total number of clusters explored in the first 10 ns of simulations by different methods and CVs. The number of clusters was calculated on nondemultiplexed trajectories from parallel tempering and parallel tempering metadynamics.

CVs and enhanced sampling methods can be evaluated based on exploration of conformational space. For this purpose, we took the first 10 ns from each simulation and calculated the cumulative number of conformational clusters sampled in the simulation (Figure 8). An unbiased MD simulation was performed for this purpose. These trajectories were analyzed using the command gmx cluster with the Gromos clustering method^46^ and the RMSD cutoff set to 0.1 nm. As expected, unbiased simulation explored the lowest number of unique conformations (clusters), namely 91. Metadynamics with ptltSNE and the combination of ptltSNE and α-RMSD explored 351 and 494 clusters, respectively. Parallel tempering and parallel tempering metadynamics explored 900 and 1215 clusters. Clustering was performed on nondemultiplexed trajectories; therefore, replicas at all temperatures contributed to sampling. In conclusion, metadynamics with ptltSNE CVs significantly enhance the exploration of the conformational space.

## Discussion

Parametric time-lagged tSNE has its advantages compared with other dimensionality reduction methods. Similarly to tSNE, it provides a very good dimensionality reduction, likely better than principal component analysis or TICA. It is difficult to compare the performance of different linear and nonlinear dimensionality reduction methods. For this purpose, we proposed a simple method. We calculated the top 20 nearest neighbors of each snapshot of the training trajectory (with 10,439 frames from 208.8 μs-trajectory) in terms of RMSD values (i.e., in high-dimensional space). Then we calculated the top 20 nearest neighbors in low-dimensional space obtained by each method. Finally, for each method, we calculated the number of common top 20 neighbors in high- and low-dimensional space. The plot (Figure S12) presents mean values for each method. Clearly, tSNE provides the best dimensionality reduction. The performance of the time-lagged tSNE is significantly lower. This is the price paid for the focus on the kinetic aspect. The performance of parametric time-lagged tSNE was comparable to that of time-lagged tSNE. The performance of PCA was lower than that of nonlinear methods. There was a drop in performance from PCA to TICA, analogous to the drop in performance from tSNE to time-lagged tSNE. In conclusion, ptltSNE provides very good dimensionality reduction while focusing on slow processes.

In this work, we selected lag time by visual inspection of ptltSNE plots constructed with different lag time values (see the Supporting Information, Figures S13–S19). The lag time should be short enough so that the analysis captures the processes of interest, but long enough to provide (close to) Markovian dynamics in the low-dimensional space. The lag time of 2 or 3 frames (40 or 60 ns) provides a good resolution of multiple long-lived states from the unfolded state. Higher lag times (80, 100, and 500 ns) provide better separation between the folded and unfolded states, but other long-lived states are poorly separated. We can speculate that simulations with a longer lag time would be better at folding the Trp-cage but worse at exploring its possible conformations.

The common practice for choosing a lag time for construction of Markov state models is by plotting implied time scales as a function of lag time.^47^ The choice can be verified using the Chapman–Kolmogorov test.^48^ Here, we cannot directly apply the same method because we dimensionally reduce trajectories but do not cluster their frames into separate microstates. Our choice of lag time (40 ns) is close to the lag time selected for the analysis of the same trajectory by two recent Markov state models (20 and 100 ns).^49,50^ Since ptltSNE is based on the same foundations as TICA, we believe that lag time was properly chosen in our study.

Another important tSNE parameter is the perplexity. Again, perplexity was selected by a trial and error approach. The results of ptltSNE with different perplexity values are presented in Supporting Information (Figures S20–S26). Low perplexity values (2–10) provide TICA-like plots with low unfolding of the high-dimensional manifold. Higher values (20–100) provide a good resolution of the analyzed structures.

Visual comparison of plots from the tSNE and time-lagged tSNE (see ref (16)) with Figure 1 indicates that the use of the neural network to map high-dimensional structures to low-dimensional space is not for free. The price paid for this is a worse separation of the individual states. Potentially, this can be addressed using a neural network with a higher number of hidden layers and neurons in each layer.

Parametric time-lagged tSNE provides a low-dimensional representation pattern similar to time-lagged tSNE.^16^ The unfolded state represents the central cluster of points (F in Figure 1). These states are characterized by RMSD values different from those of the native structures (different colors in the plot). This is clearly the effect of the time lag. The conformational changes in the unfolded state are rapid. Therefore, structures distant in RMSD are not distant when analyzed by kinetic methods such as TICA, time-lagged tSNE, or parametric time-lagged tSNE. The native structure (A), the close-to-native structure (B), and other kinetic traps (C–E) surround the unfolded state. In contrast, low-dimensional embeddings from tSNE without time lag show a relatively strong correlation with RMSD from the native structure, including for the unfolded state.^16^

As explained in the Introduction, tSNE does not attempt to reconstruct high-dimensional distances in the low-dimensional space. Instead, it reconstructs proximities. Points close in the high-dimensional space are close in the tSNE low-dimensional space. Analogously, time-lagged tSNE and ptltSNE do not reconstruct “kinetic distances.” Instead, simulation snapshots that are “kinetically close” (i.e., states with fast mutual transitions) are close in the ptltSNE low-dimensional space. For example, this is the reason behind the proximities of very unstable snapshots of the unfolded state (F in Figure 1). These snapshots are highly variable in terms of RMSD from the native structure (see the different colors of the points in cluster F in Figure 1). Because of their low stability and fast mutual transitions, they are clustered by ptltSNE.

Reconstruction of kinetic distances is provided, for example, by unsupervised machine learning in the recently introduced spectral map method.^51^ Other approaches, such as SGOOP-d^52^ or VAMP nets^53^ can also estimate the kinetic distances from simulation trajectories.

No folding was observed in the metadynamics simulation with parametric time-lagged tSNE CVs; however, we observed folding with the combination of time-lagged tSNE CVs with α-RMSD. On average, there was approximately one folding every 17 μs in the published unbiased simulation,^24^ which was used as a training data set. We observed one folding in 1.3 μs, which corresponds to 1 order of magnitude acceleration by metadynamics compared to an unbiased MD simulation. In parallel tempering metadynamics (with time-lagged tSNE CVs, without α-RMSD), we observed ten folding events in 200 ns simulation (in total 6.4 μs in all replicas). This corresponds to an acceleration of 1–2 orders of magnitude by parallel tempering metadynamics compared with an unbiased MD simulation. Furthermore, our enhanced sampling simulations explored a wider range of conformations than unbiased simulations (Figure 8).

The predicted population of the folded state (Figure 7) agrees well with the reference long simulations,^24^ where the folded state represented 25% of 208 μs at 290 K.

Folding of the Trp-cage was also studied by Meshkin and Zhu using an umbrella sampling simulation. The fraction of native contacts was used as a collective variable. Using an approximately 100 μs simulation it was possible to reconstruct the folding free energy surface in great detail.^54^

Our approach uses training data from a long unbiased simulation. Therefore, this study suffers from the “chicken-and-egg” problem, that is, it uses well-sampled trajectory as training data to enhance sampling. In our opinion, it is important to use training data that cover the relevant conformational space of the studied miniprotein but are not necessarily sampled with an accurate distribution. Metadynamics and parallel tempering metadynamics can correct for inaccuracies in the distribution of training data. Our laboratory develops and tests methods for the generation of such training data without the need to run long molecular simulations.

It is possible to address the “chicken-and-egg” problem by biased simulations, namely, by a coarse biased simulation, analysis of trajectory to obtain unbiased CVs, and recalculation of free energy surface by a biased simulation with new CVs. A similar approach was recently introduced by Rydzewski and Valsson^55^ in their multiscale reweighted stochastic embedding (MRSE) method. The method is based on parametric (not time-lagged) tSNE. The method integrates the generation of training data, construction of a Gaussian mixture probability model, and reweighting to obtain unbiased CVs. The method was tested on a model energy profile, an alanine dipeptide, and an alanine tetrapeptide. An approach based on biased sampling and CV reweighting with anisotropic diffusion maps as the dimensionality reduction method has been tested by Rydzewski and co-workers.^56^ In general, CVs obtained by unsupervised machine learning from data obtained by biased simulations are biased in terms of the density of states and geometry.^11,55−57^ This must be kept in mind when addressing the “chicken-and-egg” problem.

A problem similar to biased vs unbiased CVs is in the temperature used to obtain the training trajectory. If the simulation used to obtain CVs and the subsequent biased simulation are performed at different temperatures, then it may have an effect similar to biasing the training simulation. As far as our knowledge, this issue has not been systematically studied. We used a trajectory sampled at 290 K. The resulting CVs were applied in metadynamics at 300 K and parallel tempering metadynamics at 278–595 K. This fact must be taken into account when the results are interpreted.

It is difficult to find the reason no folding was observed in metadynamics with two ptltSNE CVs. Potential factors may include the reduced size of the neural network (because of computational constraints), the small number of ptltSNE CVs (it is possible that helix formation can be efficiently described by three CVs), or other explanations.

Addition of α-RMSD made it possible to observe one folding event in the metadynamics simulation. Figure S27 (Figure 1 colored by α-RMSD) shows that the folded state is well separated in α-RMSD as well as in the ptltSNE CVs. Therefore, it is difficult to understand why metadynamics without α-RMSD did not lead to folding. It must be kept in mind that the separation of key states is not the only requirement for an efficient collective variable. It is possible that α-RMSD captures the transition states of helix formation better than the ptltSNE CVs. On the other hand, α-RMSD is likely to accelerate only the folding and unfolding of α-helices.

In this study, we used a general measure to calculate the distance between structures (∥**x**_*i*_ – **x**_*i*_∥^2^ in eq 1), namely, mean square deviation. It is possible to use more sophisticated measures, such as smooth overlap of atomic positions (SOAPs).^58^ This would be essential in processes where the permutation of atoms is essential, for example, in simulations of phase transitions. It is also possible to use other structural data, such as interatomic distances or dihedral angles (with proper treatment of periodicity).

In this study, we demonstrated that our method could accelerate the folding of miniproteins. Taking into account the fact that fast-folding miniproteins are well studied by unbiased simulations and experimental methods and their native structures can be easily predicted by AlphaFold,^59^ we see the main potential of our CVs in conformational free energy modeling of important parts (in the size of miniproteins) of regularly sized proteins. There are many unexplored mobile elements, for example, activation loops, that control the biological activity of many proteins. The approach can also help refine some parts of proteins or to find cryptic pockets.

## Supporting Information Available

Comparison of PyTorch modules, dimensionality reduction of Trp-cage trajectory by ptltSNE colored by time, comparison of performance of different methods, ptltSNE with different values of lag time or perplexity, and ptltSNE colored by α-RMSD (PDF)
