# Supplementary material for: Acceleration of Molecular Simulations by Parametric Time-Lagged tSNE Metadynamics
Source: J Phys Chem B. 2024 Jan 18;128(4):903–13. doi: 10.1021/acs.jpcb.3c05669 (PMC10839826; doi:10.1021/acs.jpcb.3c05669)
Supplement: Supplementary file 1 — jp3c05669_si_001.pdf [file jp3c05669_si_001.pdf]

Supporting Information for Acceleration of Molecular Simulations by Parametric Time-Lagged tSNE Metadynamics

Helena Hradiská,† Martin Kurečka,‡ Jan Beránek,† Guglielmo Tedeschi,† Vladimír Višňovský,‡ Aleš Křenek,‡ and Vojtěch Spiwok*,†

†*Department of Biochemistry and Microbiology, University of Chemistry and Technology, Prague, Technická 3, Prague 6, 166 28, Czech Republic*

‡*Institute of Computer Science, Masaryk Univerzity, Šumavská 416/15, Brno, 602 00, Czech Republic*

E-mail: spiwokv@vscht.cz

Phone: +420 220 44 3028. Fax: +420 220 44 5155

Comparison of PyTorch Modules

In our work, we used our own PyTorch module available at `https://github.com/kurecka/plumed2/tree/uvt_extensions`. A new PyTorch module was introduced to Plumed by Dr. Luigi Bonati (*Istituto Italiano di Tecnologia*) at the time of completing our work. While Dr. Bonati's implementation is far more generic, and it can be used in many different scenarios, ours emphasizes performance in specific scenarios we focus on. We provide access to a PyTorch model as a bare collective variable rather than function. Instead of taking arbitrary Plumed variables (ARGs) on input, it picks ATOMS and feeds their Cartesian coordinates directly to the input layer of the TorchScript model. In this way, we can bypass lots of Plumed's interpreted processing, which makes difference when we need e.g. all heavy atoms of a large protein as the model input.

Both modules are compatible with the following differences:

- The keyword `PYTORCH_MODEL_CV` in our module is replaced by `PYTORCH_MODEL` in the current Plumed code.
- Our code uses directly atomic coordinates by the keyword:
  `PYTORCH_MODEL_CV ATOMS=1,5,13,14,7`... etc.
  with indexes of atoms. The current code in Plumed uses variables as inputs. The example above can be converted to the current code in Plumed as:

```
a1: POSITION ATOM=1 NOPBC
a2: POSITION ATOM=5 NOPBC
a3: POSITION ATOM=13 NOPBC
a4: POSITION ATOM=14 NOPBC
a5: POSITION ATOM=7 NOPBC
...
PYTORCH_MODEL ARG=a1.x,a1.y,a1.z,a2.x,a2.y,a2.z...
```

The results (values of collective variables and their derivatives are almost identical:

- Collective variables from our code:

```
#! FIELDS time ptm.node-0 ptm.node-1
 0.000000 5.642246 10.418288
 100.000000 5.605054 10.428493
 200.000000 5.268570 10.868685
 300.000000 5.250191 10.673785
 400.000000 5.979082 11.147072
 500.000000 5.784997 10.139742
```

- Collective variables from Plumed:

```
#! FIELDS time ptm.node-0 ptm.node-1
 0.000000 5.642244 10.418289
 100.000000 5.605058 10.428486
 200.000000 5.268566 10.868676
 300.000000 5.250195 10.673798
 400.000000 5.979080 11.147075
 500.000000 5.785001 10.139745
```

- Derivatives from our code:

```
#! FIELDS time parameter ptm.node-0 ptm.node-1
 0.000000 0         -1.1854         -1.9170
 0.000000 1          0.0918          1.7600
 0.000000 2          0.0624          0.5827
 0.000000 3          0.2509          0.4997
 0.000000 4         -0.2232         -1.6264
 0.000000 5         -0.3299         -0.8539
```

- Derivatives from Plumed:

```
#! FIELDS time parameter ptm.node-0 ptm.node-1
 0.000000 0         -1.1854         -1.9170
 0.000000 1          0.0918          1.7600
 0.000000 2          0.0624          0.5827
 0.000000 3          0.2509          0.4997
 0.000000 4         -0.2232         -1.6264
 0.000000 5         -0.3299         -0.8539
```

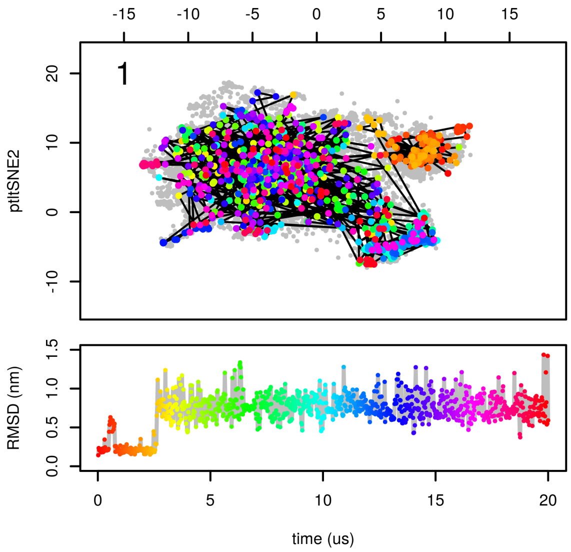

Figure S1: Dimensionality reduction of Trp-cage trajectory by ptltSNE colored by time (0-20 μs).

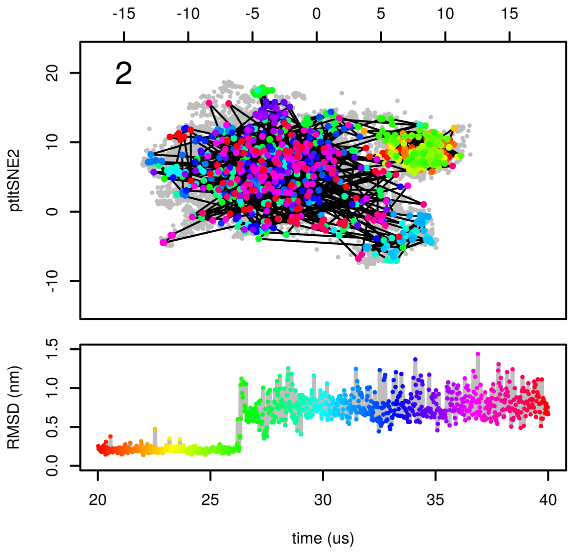

Figure S2: Dimensionality reduction of Trp-cage trajectory by ptltSNE colored by time (20-40 μs).

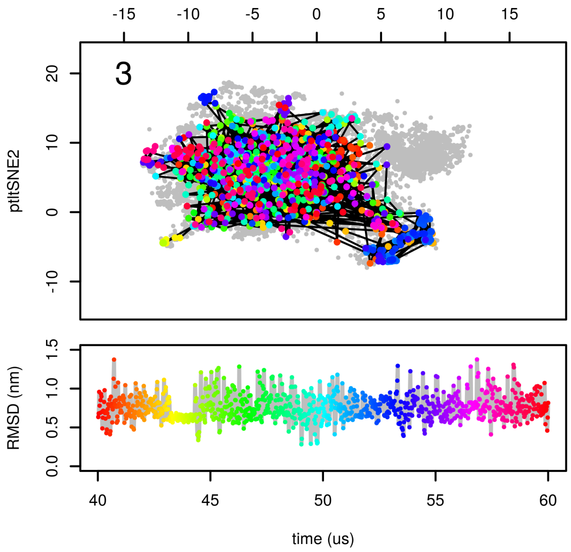

Figure S3: Dimensionality reduction of Trp-cage trajectory by ptltSNE colored by time (40-60 μs).

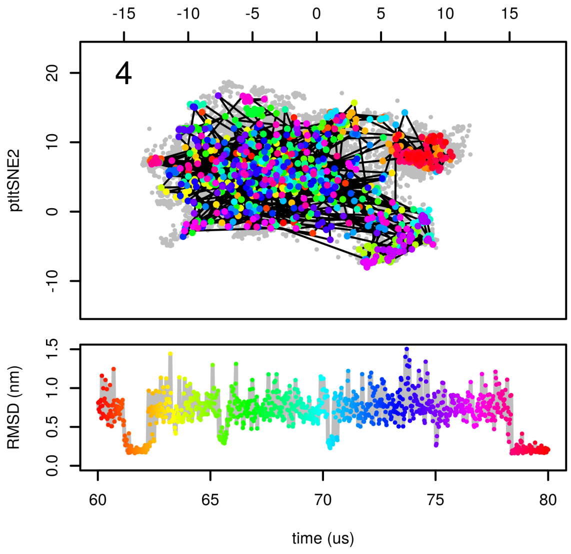

Figure S4: Dimensionality reduction of Trp-cage trajectory by ptltSNE colored by time (60-80 μs).

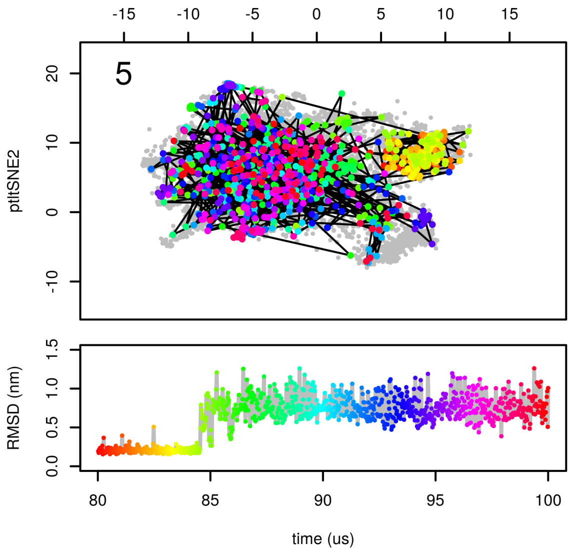

Figure S5: Dimensionality reduction of Trp-cage trajectory by ptltSNE colored by time (80-100 μs).

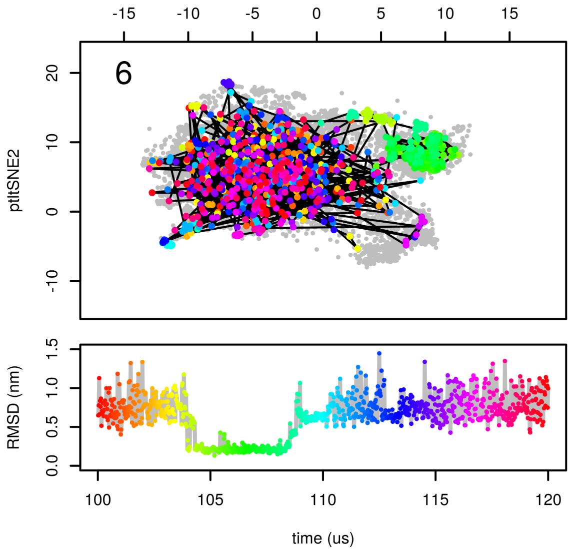

Figure S6: Dimensionality reduction of Trp-cage trajectory by ptltSNE colored by time (100-120 μs).

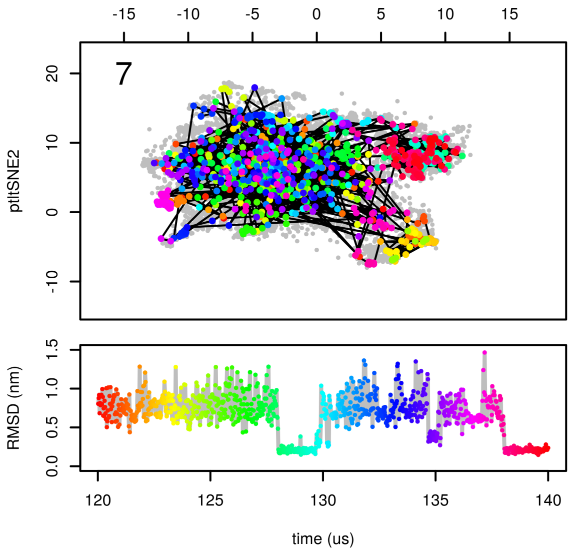

Figure S7: Dimensionality reduction of Trp-cage trajectory by ptltSNE colored by time (120-140 μs).

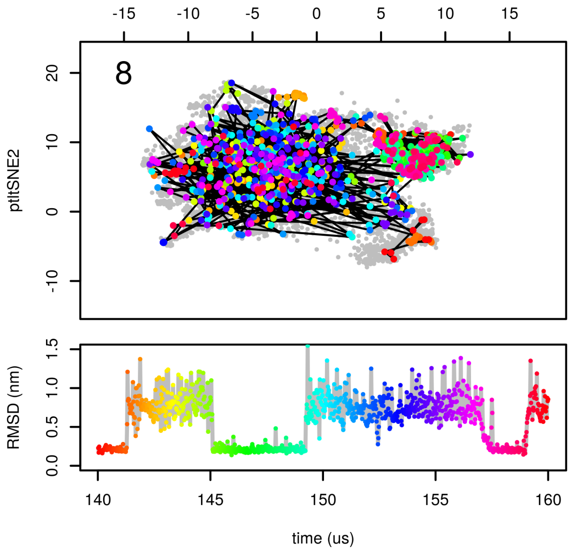

Figure S8: Dimensionality reduction of Trp-cage trajectory by ptltSNE colored by time (140-160 μs).

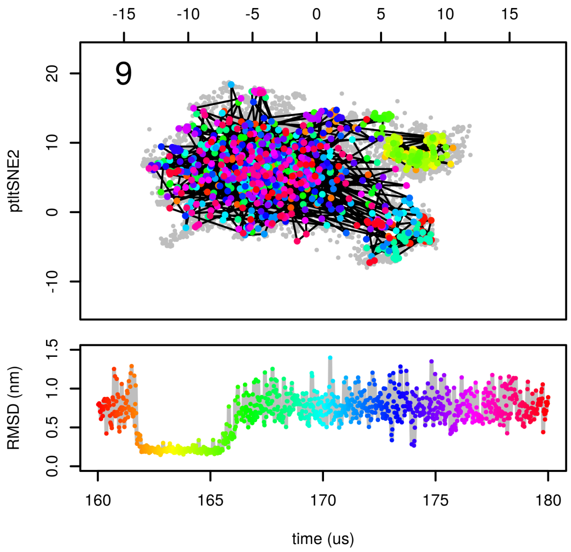

Figure S9: Dimensionality reduction of Trp-cage trajectory by ptltSNE colored by time (160-180 μs).

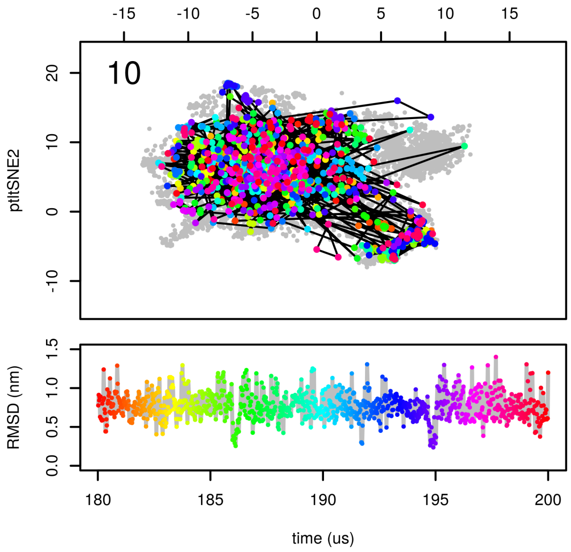

Figure S10: Dimensionality reduction of Trp-cage trajectory by ptltSNE colored by time (180-200 μs).

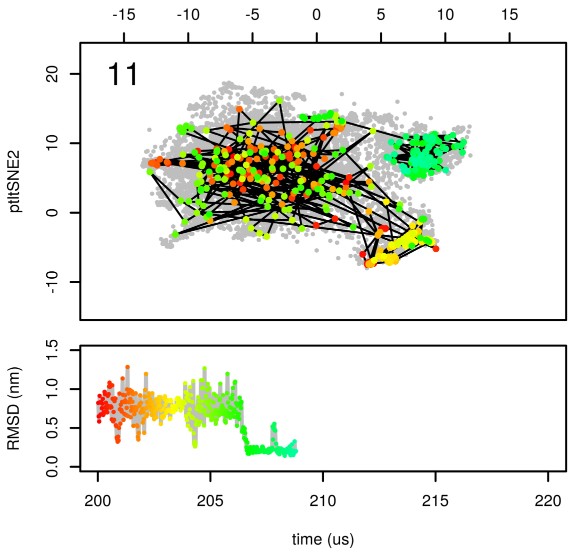

Figure S11: Dimensionality reduction of Trp-cage trajectory by ptltSNE colored by time (200-208 μs).

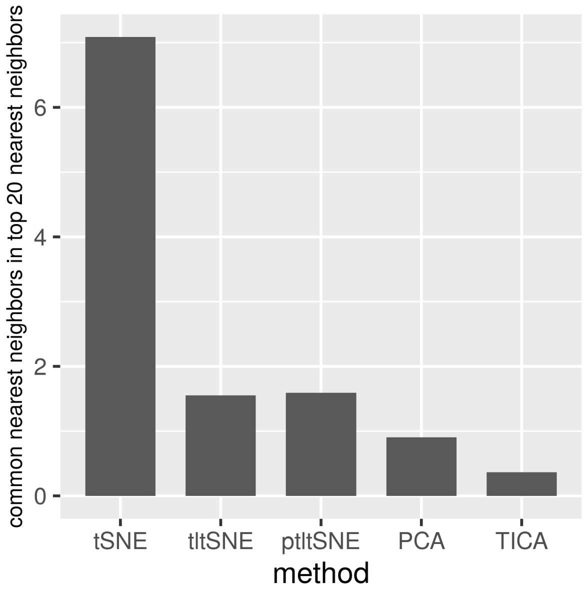

Figure S12: Comparison of performance of different dimensionality reduction methods. For each of 10440 snapshots of Trp-cage trajectory we calculated the 20 nearest neighbor snapshots in terms of RMSD. Next, we calculated the 20 nearest neighbor snapshots in a low-dimensional space. The mean number of common nearest neighbors is depicted in the plot.

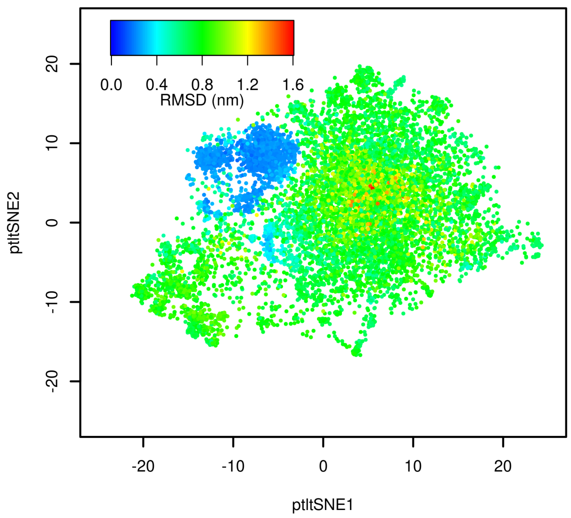

Figure S13: Dimensionality reduction of Trp-cage trajectory by ptltSNE with lag time 1 frame (20 ns) colored by RMSD.

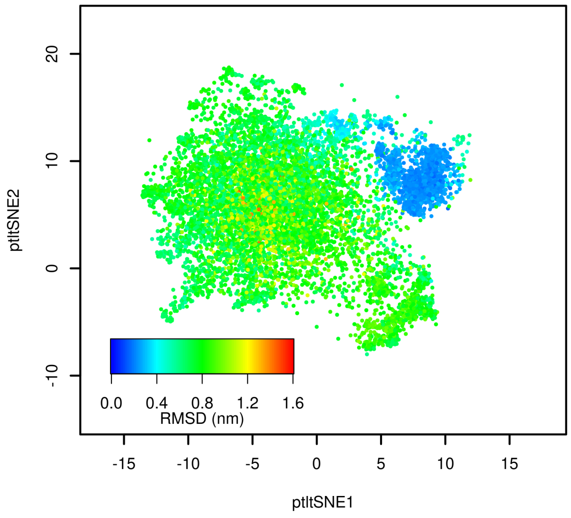

Figure S14: Dimensionality reduction of Trp-cage trajectory by ptltSNE with lag time 2 frames (40 ns) colored by RMSD (same as in Figure 1).

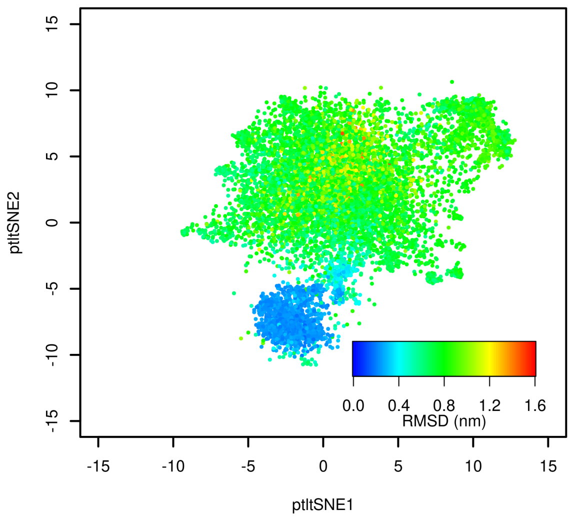

Figure S15: Dimensionality reduction of Trp-cage trajectory by ptltSNE with lag time 3 frames (60 ns) colored by RMSD.

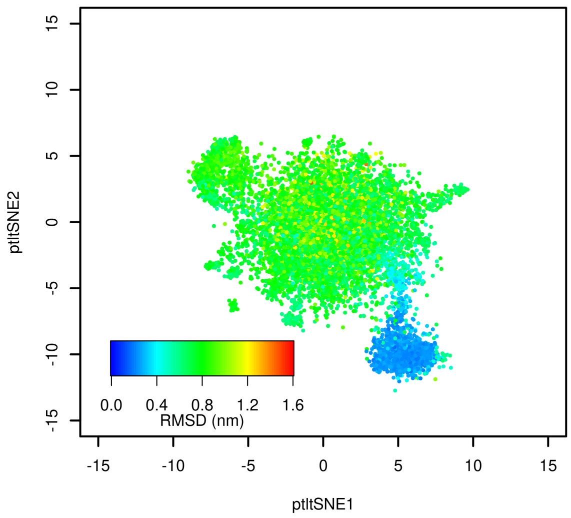

Figure S16: Dimensionality reduction of Trp-cage trajectory by ptltSNE with lag time 4 frames (80 ns) colored by RMSD.

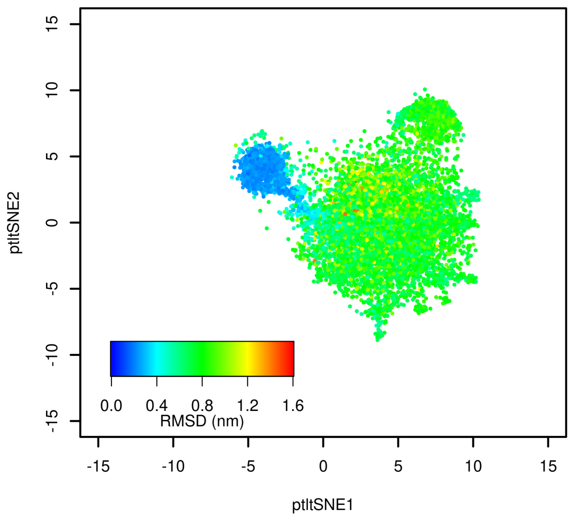

Figure S17: Dimensionality reduction of Trp-cage trajectory by ptltSNE with lag time 5 frames (100 ns) colored by RMSD.

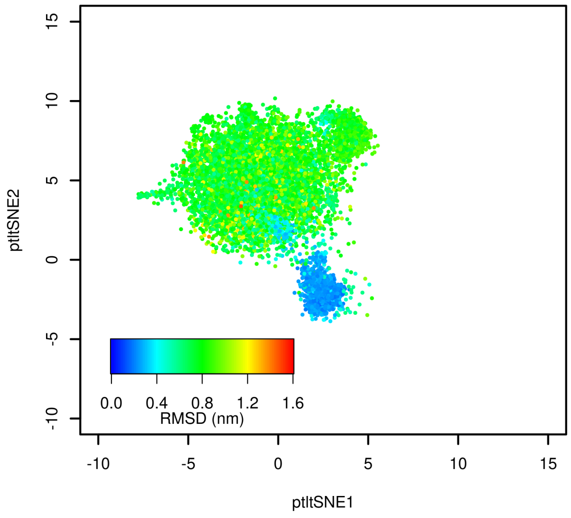

Figure S18: Dimensionality reduction of Trp-cage trajectory by ptltSNE with lag time 10 frames (200 ns) colored by RMSD.

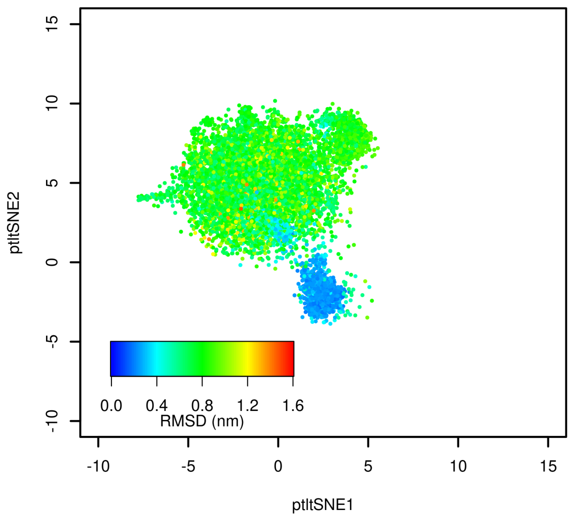

Figure S19: Dimensionality reduction of Trp-cage trajectory by ptltSNE with lag time 10 frames (200 ns) colored by RMSD.

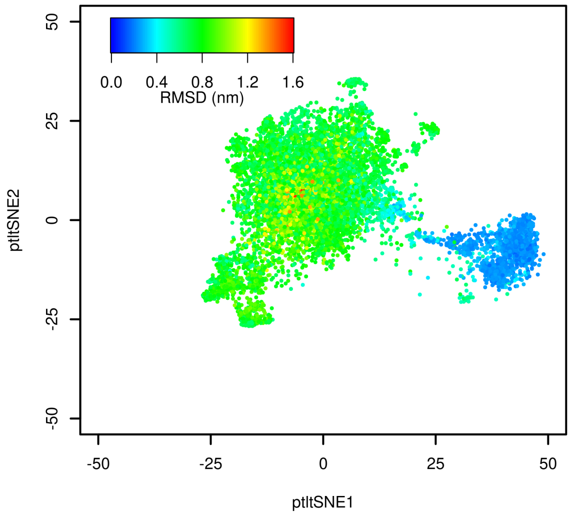

Figure S20: Dimensionality reduction of Trp-cage trajectory by ptltSNE with perplexity set to 2 colored by RMSD.

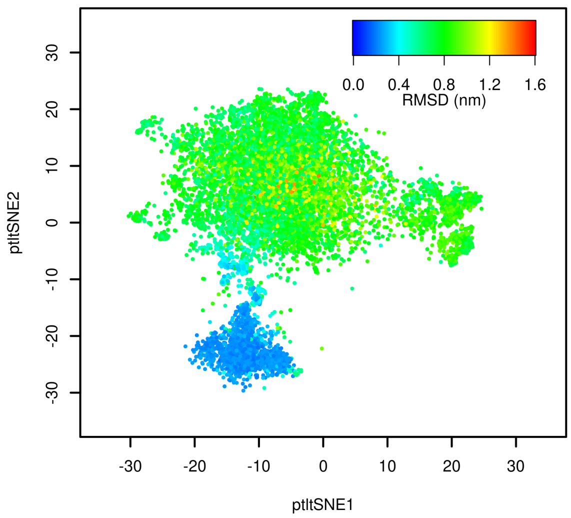

Figure S21: Dimensionality reduction of Trp-cage trajectory by ptltSNE with perplexity set to 5 colored by RMSD.

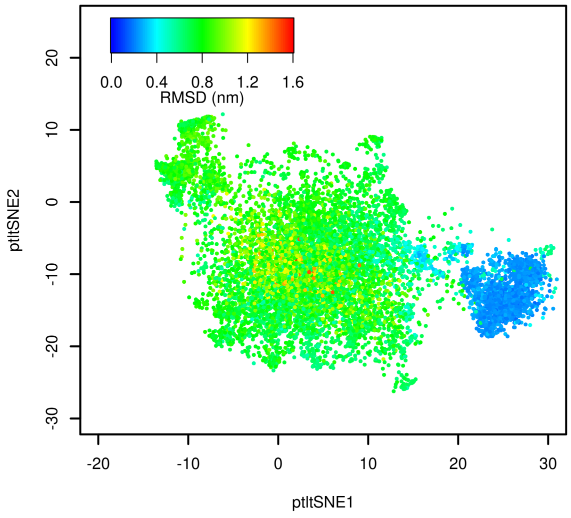

Figure S22: Dimensionality reduction of Trp-cage trajectory by ptltSNE with perplexity set to 10 colored by RMSD.

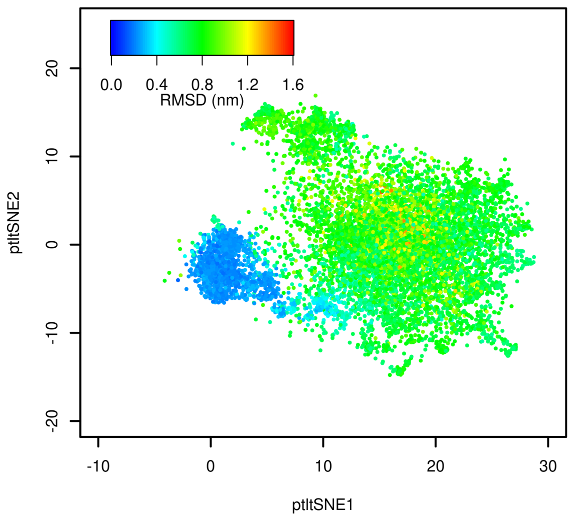

Figure S23: Dimensionality reduction of Trp-cage trajectory by ptltSNE with perplexity set to 20 colored by RMSD.

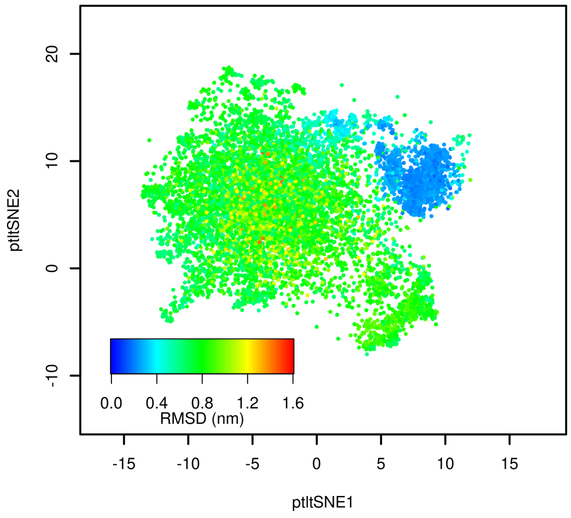

Figure S24: Dimensionality reduction of Trp-cage trajectory by ptltSNE with perplexity set to 30 colored by RMSD (same as in Figure 1).

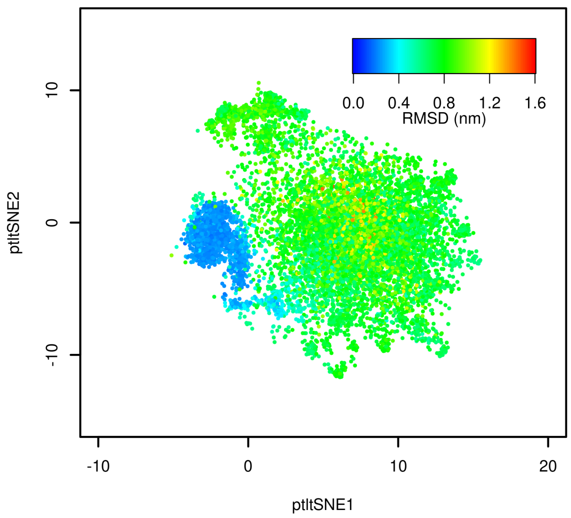

Figure S25: Dimensionality reduction of Trp-cage trajectory by ptltSNE with perplexity set to 50 colored by RMSD.

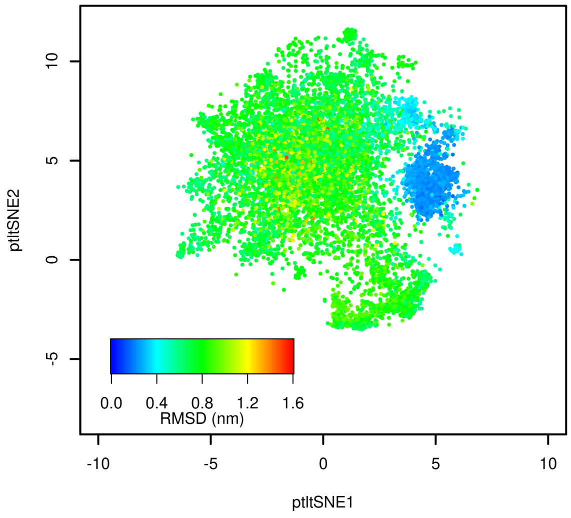

Figure S26: Dimensionality reduction of Trp-cage trajectory by ptltSNE with perplexity set to 100 colored by RMSD.

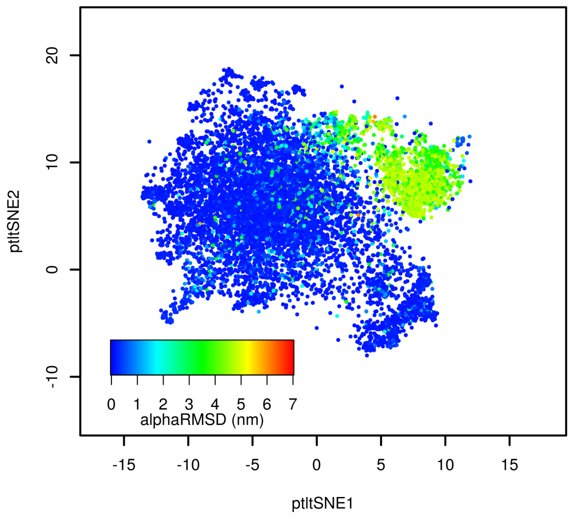

Figure S27: Dimensionality reduction of Trp-cage trajectory by ptltSNE colored by α-RMSD.
